# AFM Imaging Reveals Multiple Conformational States of ADAMTS13

**DOI:** 10.1186/s13036-018-0102-y

**Published:** 2019-01-22

**Authors:** Shanshan Yu, Wang Liu, Jinhua Fang, Xiaozhong Shi, Jianhua Wu, Ying Fang, Jiangguo Lin

**Affiliations:** Institute of Biomechanics, School of Biosciences and Bioengineering, South China University of Technology, Guangzhou, 510006 China

**Keywords:** ADAMTS13, atomic force microscopy, conformational states, pH

## Abstract

**Background:**

ADAMTS13 (A disintegrin and metalloprotease with a thrombospondin type 1 motif 13) cleaves Von Willebrand factor (VWF) to regulate its size, thereby preventing aberrant platelet aggregation and thrombus. Deficiency of ADAMTS13 caused by either genetic mutations or by inhibitory autoantibodies against ADAMTS13 leads to thrombotic thrombocytopenic purpura (TTP). Recently, ADAMTS13 was reported to adopt a “closed” conformation with lower activity and an “open” one resulting from the engagements of VWF D4-CK domains or antibodies to the distal domains of ADAMTS13, or mutations in its spacer domain. These engagements or mutations increase ADAMTS13 activity by ~ 2.5-fold. However, it is less known whether the conformation of ADAMTS13 is dynamic or stable.

**Results:**

Wild type ADAMTS13 (WT-ADAMTS13) and the gain-of-function variant (GOF-ADAMTS13) with five mutations (R568K / F592Y / R660K / Y661F / Y665F) in spacer domain were imaged by atomic force microscopy (AFM) at pH 6 and pH 7.5. The data revealed that at both pH 6 and pH 7.5, WT-ADAMTS13 adopted two distinct conformational states (state I and state II), while an additional state (state III) was observed in GOF-ADAMTS13. In the present study, we propose that state I is the “closed” conformation, state III is the “open” one, and state II is an intermediate one. Comparing to pH 7.5, the percentages of state II of WT-ADAMTS13 and state III of GOF-ADAMTS13 increased at pH 6, with the decrease in the state I for WT-ADAMTS13 and state I and state II for GOF-ADAMTS13, suggesting lower pH extended the conformation of ADAMTS13.

**Conclusion:**

Both WT- and GOF-ADAMTS13 exist multiple conformational states and lower pH might alter the tertiary structure and/or disrupt the intra-domain interactions, increasing the flexibility of ADAMTS13 molecules.

**Electronic supplementary material:**

The online version of this article (10.1186/s13036-018-0102-y) contains supplementary material, which is available to authorized users.

## Background

The enzyme ADAMTS13 (A disintegrin and metalloprotease with a thrombospondin type 1 motif 13) specifically cleaves the peptide bond Tyr1605-Met1606 in A2 domain of Von Willebrand factor (VWF) to mediate its size, downregulating the prothrombotic activity of VWF[1–3]. Deficiency of ADAMTS13 activity, caused by either mutations in the ADAMTS13 gene or by inhibitory autoantibodies against ADAMTS13, results in the accumulation of ultra-large VWF (ULVWF) in plasma, leading to excessive platelet aggregation and disseminated VWF/platelet-rich thrombus formation, which is the characteristic feature of thrombotic thrombocytopenic purpura (TTP)[1, 4–7]. Mild to moderate deficiency of ADAMTS13 activity or increased ratios of VWF to ADAMTS13 have been shown to be risk factors for the development of systemic inflammation, myocardial or cerebral infarction, preeclampsia or eclampsia, and cerebral malaria[8–11].

ADAMTS13 is synthesized and released from hepatic stellate cells, endothelial cells, megakaryocytes and platelets[1, 12]. A mature ADAMTS13 is composed of a metalloprotease domain (M), a disintegrin domain (D), a thrombospondin type-1 motif (TSP-1, T), a cysteine-rich domain (C), a spacer (S), seven additional TSP-1 motifs, and two CUB (complement components C1r/C1s, epidermal growth factor-related sea urchin protein, and bone morphogenetic protein-1) domains[2]. The two CUB domains are unique for ADAMTS13[2]. Recently, ADAMTS13 was shown to adopt a “closed” conformation through the binding between its spacer and CUB domains[13, 14]. Binding of the VWF C-teriminal D4-CK or antibodies to CUB domains disrupts the spacer-CUB interaction, resulting in an “open” conformation, leading to ~ 2.5-fold increase in ADAMTS13 activity[13–15]. Five amino acids (R568, F592, R660, Y661, Y665) in spacer domain were reported to be responsible for the spacer-CUB interaction. The gain-of-function (GOF) ADAMTS13 variant, in which the five amino acids are mutated, does not bind to the fragment of CUB domains in solution[13]. Three linkers in ADAMTS13 distal domains increase the protein flexibility and facilitate the interaction between spacer and CUB domains[16]. However, the “open” ADAMTS13 is capable of proteolysing the Aα chain of fibrinogen, suggesting conformational activation of ADAMTS13 might induce proteolytic promiscuity[17].

In addition to conformational activation, pH was reported to change the activity of ADAMTS13 as well. ADAMTS13 is most active at pH 6, with markedly reduced at physiological pH[14, 18–20]. This phenomenon has been attributed to ionization of a Zn^2+^-bound water molecule in the active site[14, 20]. However, the observation that the proximal variant of ADAMTS13 (MDTCS) has comparable activity in the various pH ranging from 6 to 7.4, suggests that titration of the active site or the substrate is not the reason for the pH dependency of ADAMTS13 activity[14]. The authors proposed that the protonation of specific residues at pH 6 alters critical interactions between distal and proximal ADAMTS13 domains leading to an “open” conformation; this, in turn, increases the ADAMTS13 activity[14].

It is well established that ADAMTD13 is able to form a “closed” conformation and an “open” one upon activation. However, without activation, “open” form of ADAMTS13 was detected in solution as well[21]. It is not clear whether the conformation of ADAMTS13 is stable or dynamic in circulation. To address this question, wild-type ADAMTS13 (WT-ADAMTS13) and GOF-ADAMTS13 molecules were imaged by atomic force microscopy (AFM) at pH 6 or pH 7.5. We found that WT-ADAMTS13 molecules adopted two states, while GOF-ADAMTS13 molecules adopted three states at both pH conditions. Lower pH induced ADAMTS13 molecules to shift from condensed to elongated conformation.

## Methods

### Reagents

Recombinant WT-ADAMTS13 plasmid with a C-terminal 6*His-tag in pSecTag2A vector containing XhoI and HindIII restriction sites was a generous gift from Jing-Fei Dong (BloodWorks Northwest Research Institute, Seattle, WA USA). Recombinant GOF-ADAMTS13 plasmid with five mutations (R568K/F592Y/R660K/Y661F/Y665F), was constructed by site-directed mutagenesis with QuikChange II XL Site-Directed Mutagenesis kit (Agilent, California, USA) according to the manufacturer’s instructions with the WT-ADAMTS13 plasmid as a template. Restriction enzymes XhoI and HindIII-HF were purchased from NEB (Ipswich, MA, USA). HEK293T cells were purchased from Life Technologies (Grand Island, NY, USA). Recombinant Human VWF-A2 and commercial ADAMTS13 protein were purchased from R & D Systems (Minneapolis, MN, USA). The plasmid of VWF-A1 was a generous gift from Miguel A. Cruze (Baylor College of Medicine, Houston, TX, USA). Cell culture media Dulbecco’s Modified Eagle Medium (DMEM), improved Minimal Essential Medium (Opti-MEM) and fetal bovine serum (FBS) were purchased from GIBCO™ (Grand Island, NY, USA). Lipofectamine 3000 was purchased from Invitrogen™ (Waltham, MA, USA). Rabbit anti-His monoclonal antibody and goat anti-rabbit IgG monoclonal antibody were purchased from Abcam (Massachusetts, US). Bovine serum albumin (BSA), SuperSignal® West Pico luminescent substrate, bicinchoninic acid (BCA) Protein Assay Kit were purchased from Thermo Scientific™ (Waltham, MA, USA).

### Expression and purification of recombinant WT- and GOF-ADAMTS13

WT- and GOF-ADAMTS13 were expressed in HEK293T cells with lipofectamine 3000. Plasmid and liposome volume ratio was 1:1.5. HEK293T cell complete medium was DMEM medium with 10% FBS. The transfected cells was incubated for 2 days in 5% CO_2_ at 37°C, then screened with 1% hygromycin B (Solarbio Science & Technology, Beijing, CN). Stably transfected cell lines were obtained after about 20 days, and expanded to collect 500 ml of culture medium for purification. The proteins of interest were eluted with different concentrations of imidazole using an AKTA protein purification system (GE Healthcare) in conjunction with a Ni^2+^-Hi-Trap-chelating column (GE Healthcare). Gel filtration chromatography was performed to further purify the proteins with the Superdex™ 200 Increase 5/150 GL column (GE Healthcare). Purified proteins were verified by 7.5% SDS-PAGE (sodium dodecyl sulfate polyacrylamide gel electrophoresis) and Western blot. Protein concentration was measured with BCA kit according to the manufacturer’s instructions.

### AFM imaging experiments

Proteins were scanned with the CSPM5500 AFM system (BenYuan, Guangzhou, CN). To prepare the samples, all solutions were filtered with a 0.22 μm Milli-Q filter (Millipore, Billerica, MA). Protein of interest (WT-ADAMTS13 or GOF-ADAMTS13) was diluted with AFM buffer (25 mM NaOAc, 25 mM HEPES, pH = 7.5 / 6.0) to the final concentration of 30 nM. 30 μl of protein solution was deposited onto a cleaned mica as previously described[22, 23] for 5 min, rinsed with distilled water three times, and dried with nitrogen gas. Samples were scanned with tapping mode by using the PPP-FMR-20 AFM probe (NANOSENSER™, resonance frequency of 45–115 Hz, spring constant of 0.5–9.5 N/m). The scanning range was 1 μm × 1 μm, with the resolution of 512 line × 512 line, and scanning frequency of 1.5 Hz.

### AFM adhesion experiments

To carry out the adhesion experiments, the CSPM5500 system was modified in our laboratory. Briefly, the built-in feedback control loop was shut down and an external feedback control loop was added which was achieved by Labview (NI, USA) programing to control the piezoelectric translator and collect data through a data acquisition card (SCB-68A, NI, USA). The procedure of AFM tip functionalization was the same as previously described[3]. Each AFM tip (Bruker MLCT) was soaked in 15 μl of VWF-A2 (13 μg/mL) or phosphate buffered saline (PBS, GIBCO) containing 2% BSA at 4 °C overnight, followed by being soaked in PBS containing 2% BSA to block nonspecific binding for 30 min on the second day. Polystyrene dishes were cleaned with absolute ethanol and dried with nitrogen gas before protein adsorption. Surfaces were incubated with 15 ul per spot of WT-ADAMTS13 or GOF-ADAMTS13 (15 μg/ml) at 4 °C overnight and washed 3 times with PBS, then 2.5 ml of PBS containing 2% BSA was added to the dish and incubated for 30 min at room temperature to reduce nonspecific adhesion. The spring constant of each tip was calibrated by thermal fluctuation method[24]. The spring constant of the tips ranges from 15 to 30 pN/m which is in the reasonable range according to manufacturer’s instruction. During each measurement cycle, the functionalized (VWF-A2) or non-functionalized (2% BSA, bared) tip was brought to contact a spot (protein of interested coated or 2% BSA coated) for 0.5 s followed by being retracted to the initial position. An event that the voltage signal significantly increased during retraction was considered as an adhesive event. 100 cycles were repeated at each spot and the adhesion frequency was calculated. More than 3 different spots were detected for each sample which was considered as valid when the adhesion frequency of nonspecific interaction was less than 5%, the same criterion as previously described[3].

### Data analysis

All AFM images were processed with Imager4.7.23 or Gwyddion2.43 software to derive parameters (volume, projected area, maximum length and aspect ratio) for each molecule. To exclude the aggregated molecules, only those molecules whose volume was in 95% confidence interval of the first peak (μ ± 1.96σ, Additional file 1 Figure. S1) were selected for further analysis.

For statistics, Student’s t-test was performed to test the difference in adhesion frequency and One-way ANOVA test was performed to test the difference in volume, projected area, maximum length and aspect ratio. Data were presented as mean ± SD. The *p*-values below 0.01 were deemed statistically significant and denoted by *. ** denotes *p* < 0.001.

## Results

### Proteins were successfully imaged by AFM scanning

**Fig. 1:**
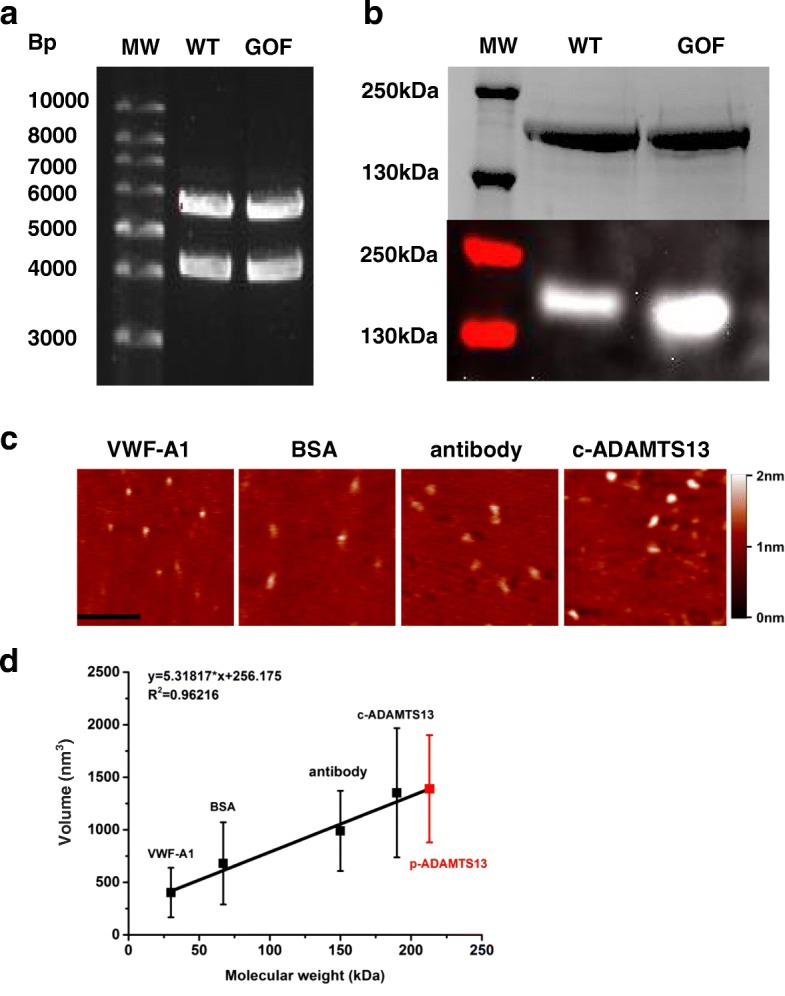
AFM system successfully imaged proteins**.** (**a**) WT- and GOF-ADAMTS13 plasmids were double digested by restriction enzymes HindIII and XhoI, and verified in agarose gel electrophoresis. (**b**) Purified WT- and GOF-ADAMTS13 were analyzed by SDS-PAGE under reducing conditions on a 7.5% gel and Western blotting with anti-His antibody. (**c**) AFM images of VWF-A1 (30 kDa), BSA (67 kDa), anti-His tag antibody (150 kDa) and commercial ADAMTS13 (190 kDa). The horizontal scale bar is 200 nm. The vertical scale bar indicates the height. (**d**) The plot of volumes of these four proteins versus their molecular weights. The data was well fitted into a straight line. The goodness of fit is indicated by R^2^. According to the linear relationship y = 5.31817*x + 256.175, the volume of purified WT-ADAMTS13 (red square) corresponds to the molecular weight of 213 kDa. Data were presented as mean ± SD.

Recombinant WT-ADAMTS13 plasmid served as a template to generate the plasmid of GOF-ADAMTS13 with five mutations (R568K/F592Y/R660K/Y661F/Y665F, Methods). The plasmid of GOF-ADAMTS13 was sequenced and compared to the full-length human ADAMTS13 gene sequence in the NCBI database (Gen Bank Accession No. NM_139025.4), confirming that the five mutations were successfully induced and no other mutations were induced (Data not shown). To verify the plasmids of WT-ADAMTS13 and GOF-ADAMTS13, restriction enzymes XhoI and HindIII were used to digest the plasmids and analyzed by agarose gel electrophoresis (Methods). Two bands at 5700 bp and 4280 bp were observed for each type of plasmid, corresponding to the vector and WT- or GOF-ADAMTS13 fragment respectively as expected (Fig. 1a). WT- and GOF-ADAMTS13 were expressed in HEK293T cell, purified with Ni^2+^-Hi-Trap-chelating column and further purified with gel filtration chromatography (Methods). SDS-PAGE analysis clearly showed a band between 130 kDa and 250 kDa for WT-ADAMTS13 or GOF-ADAMTS13, consistent with the apparent molecular weight of ADAMTS13 which is ~ 190 kDa[25] (Fig. 1b). Western blotting analysis by using the anti-His antibody targeting His-tag in the C-termini of WT- or GOF-ADAMTS13 further confirmed that the proteins of interest were successfully expressed and purified (Fig. 1b). The purity of WT-ADAMTS13 or GOF-ADAMTS13 was higher than 90%, determined by Coomassie Blue staining.

To verify the AFM system in protein imaging, four different sizes of proteins, VWF-A1 (30 kDa), BSA (67 kDa), anti His-tag antibody (150 kDa) and commercial WT-ADAMTS13 (190 kDa) were imaged (Fig. 1c). The average volume of VWF-A1, BSA, His-tag antibody and commercial WT-ADAMTS13 were 402.8 ± 236.0 nm^3^ (*n* = 110), 680.5 ± 391.1 nm^3^ (*n* = 125), 990.0 ± 381.2 nm^3^ (*n* = 122) and 1352.5± 615.2 nm^3^ (*n* = 229), respectively. The plot of average volume of these four molecules versus molecular weight was well fit into a straight line (Fig. 1d), indicating that the measured volume are well correlated to molecular weight of a protein, and that AFM system is capable of imaging protein successfully[26, 27].

### Purified ADAMTS13 were correctly folded and functional

**Fig. 2:**
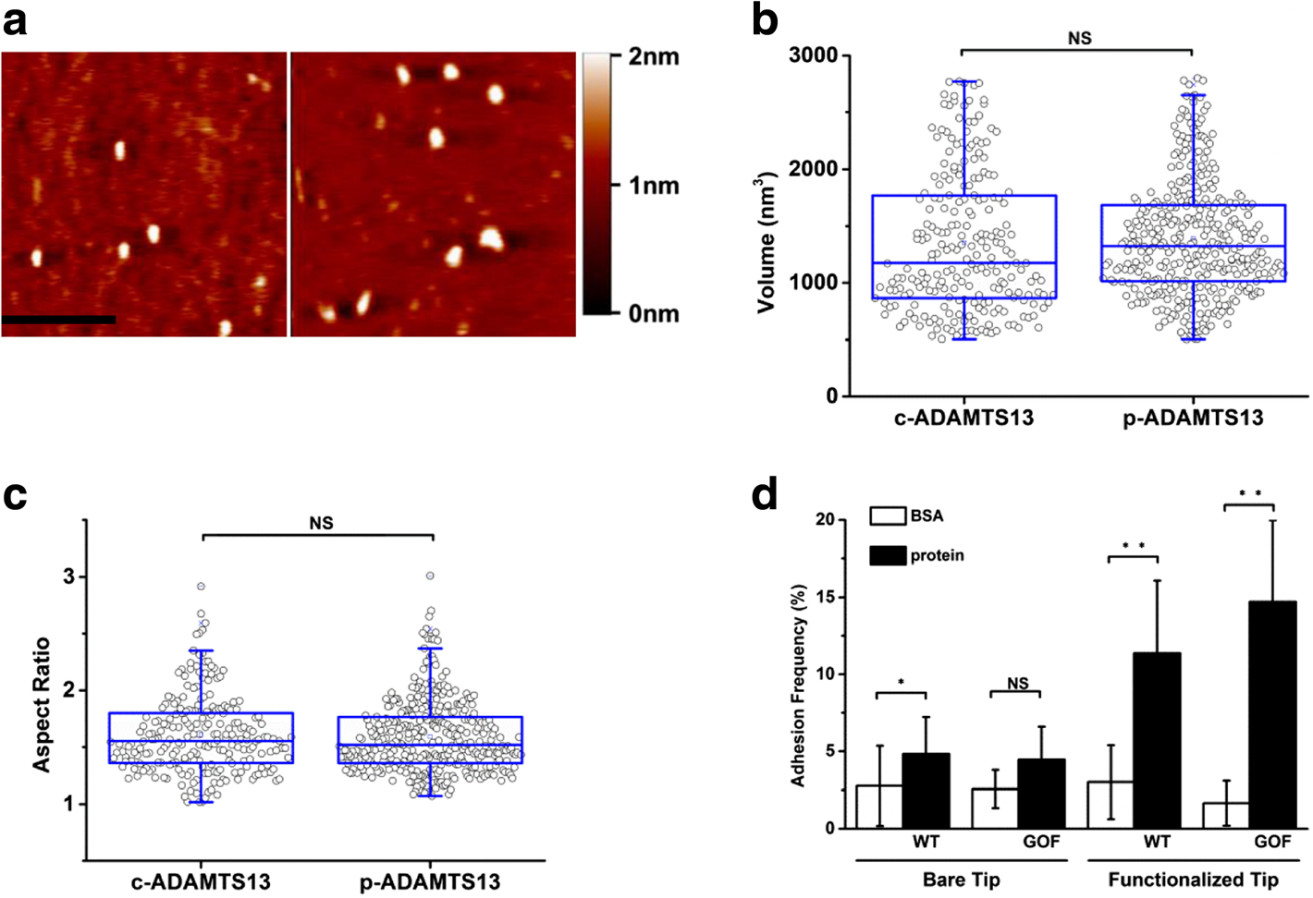
Purified ADAMTS13 were correctly folded and functional. (**a**) AFM images of purified WT-ADAMTS13 (left) and commercial WT-ADAMTS13 (right). The horizontal scale bar is 200 nm. The vertical scale bar indicates the height. The volume (**b**) and aspect ratio (**c**) of purified WT-ADAMTS13 (*n* = 229) and commercial WT-ADAMTS13 (*n* = 369) at pH 7.5. (**d**) WT- and GOF-ADAMTS13 specifically bound to VWF-A2. The experiments of bared or functionalized AFM tips contacting to BSA coated surfaces served as controls (Hollow bars). The left two black bars were the adhesion frequencies of bared tips contacting to the WT- or GOF-ADAMTS13 coated surfaces. The right two black bars were the adhesion frequencies of VWF-A2 functionalized AFM tips contacting to the WT- or GOF-ADAMTS13 coated surfaces. Data were presented as mean ± SD.

To confirm that the purified ADAMTS13 molecules folded correctly, purified WT-ADAMTS13 was compared with commercial WT-ADAMTS13 (Fig. 2a). The volume of purified WT-ADAMTS13 was 1390.3 ± 509.7 nm^3^, corresponding to the molecular weight of 213 kDa (Fig. 1d, red square), based on the linear relationship between volume and molecular weight. Although the estimated molecular was slightly higher than the one previously reported[25], no significant difference was observed between purified and commercial WT-ADAMTS13 in both volume (1390.3 ± 509.7 nm^3^ vs 1352.5± 615.2 nm^3^ on average, Fig. 2b) and aspect ratio (1.59 ± 0.32 vs 1.60 ± 0.34 on average, Fig. 2c), indicating the purified ADAMTS13 molecules folded correctly.

To verify the biological functions of recombinant WT- and GOF-ADAMTS13, the binding frequencies of WT- and GOF-ADAMTS13 to VWF-A2 were measured by AFM. The adhesion frequencies of bare tips or VWF-A2 functionalized tips contacting to BSA coated spots (Fig. 2d hollow bars, *n* = 15 for each condition) and the ones of bare tips contacting to WT- or GOF-ADAMTS13 coated spots (Fig. 2d, the 1st and 2nd black bars, *n* = 25 for each condition) were ~ 4%, indicating that 2% BSA effectively blocked the nonspecific adhesion between the tips and the dish surfaces. In contrast, the specific binding frequencies of VWF-A2 functionalized tips to ADAMTS13 coated spots were 11.37 ± 4.71% (*n* = 27) for WT and 14.70 ± 5.27% (*n* = 30) for GOF (Fig. 2d, the 3rd and 4th black bars), respectively, significantly higher than nonspecific ones.

Together, the data demonstrated that ADAMTS13 molecules were successfully purified, correctly folded and biologically functional.

### ADAMTS13 molecules adopted multiple conformational states

**Fig. 3:**
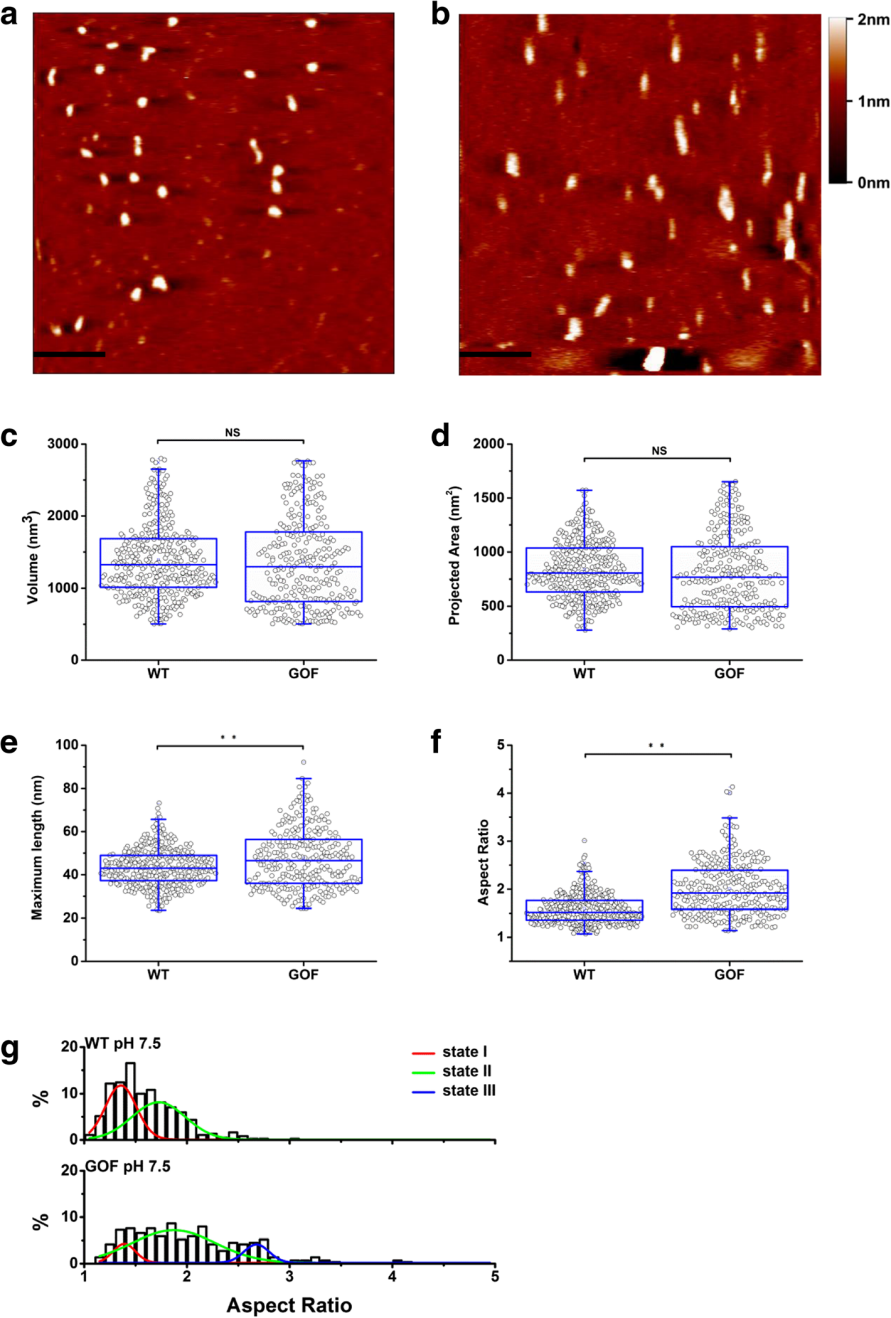
WT- and GOF-ADAMTS13 adopted multiple conformational states. AFM images of purified WT-ADAMTS13 (**a**) and GOF-ADAMTS13 (**b**) at pH 7.5. The horizontal scale bar is 200 nm. The vertical scale bar indicates the height. Volume (**c**), projected area (**d**) maximum length (**e**) and aspect ratio distribution (**f**) analysis of WT-ADAMTS13 (n = 369) and GOF-ADAMTS13 (*n* = 289) at pH 7.5. (**g**) The histogram of aspect ratio of WT-ADAMTS13 and GOF-ADAMTS13. The histogram of WT-ADAMTS13 was well fitted with two Gaussian distributions. The histogram of GOF-ADAMTS13 was well fitted with three Gaussian distributions.

Previous studies reported that two CUB domains of WT-ADAMTS13 bind to the spacer domain to form a “closed” conformation, while the disruption of the CUB-spacer interaction results in ~ 2.5-fold increase in proteolytic activity[13–16]. However, it is still not clear whether the conformations of WT- and GOF-ADAMTS13 are dynamic or stable in solution. To address this question, purified WT- and GOF-ADAMTS13 molecules pre-treated at pH 7.5 were deposited on clean mica surfaces and imaged with AFM (Fig. 3a and b). The volume (Fig. 3c), projected area (Fig. 3d), maximum length (Fig. 3e) and aspect ratio (Fig. 3f) of WT- and GOF-ADAMTS13 molecules were analyzed. The volumes of WT- and GOF-ADAMTS13 (1390.3 ± 509.7 nm^3^ vs 1357.8 ± 627.1 nm^3^ on average) were not significantly different as expected (Fig. 3c). In addition, no significant difference on projected area was observed between WT- and GOF-ADAMTS13 neither (835.6 ± 264.7 nm^2^ vs 801.9 ± 355.9 nm^2^ on average, Fig. 3d). The average maximum length of GOF-ADAMTS13 was 47.6 ± 13.7 nm, significantly longer than the one of WT-ADAMTS13 (43.4 ± 8.7 nm, Fig. 3e). The difference of aspect ratio between WT- and GOF-ADAMTS13 was significant as well (1.59 ± 0.32 vs 2.01 ± 0.56 on average, Fig. 3f).

To further analyze the conformational states of ADAMTS13, the distributions of aspect ratio of WT- and GOF-ADAMTS13 molecules were plotted (Fig. 3g). Comparing to the distribution of WT-ADAMTS13 which ranged from 1 to 3.2, the one of GOF-ADAMTS13 was broader, from 1 to 4.8, indicating more molecules adopted “open” conformation in GOF-ADAMTS13 group, consistent with previous reports[13, 14]. Interestingly, the aspect ratio of WT-ADAMTS13 exhibited two Gaussian distributions, but not a single one, indicating WT-ADAMTS13 adopts two distinct conformational states (state I and state II, Fig. 3g). The center of the state I was 1.36 ± 0.02 with the percentage of 45.81% (Fig. 3g), while the center of the state II was 1.73 ± 0.12 with the percentage of 54.19% (Fig. 3 g). In contrast, an additional conformational state (state III, center at 2.68 ± 0.03) was observed in the aspect ratio distribution of GOF-ADAMTS13 (Fig. 3g). Comparing to the ones of WT-ADAMTS13, the percentage of state I of GOF-ADAMTS13 reduced 34.03% (from 45.81 to 11.78%, Table 1), while the one of state II increased 21.17% (from 54.19 to 75.36%, Table 1). The percentage of state III was 12.86% (Table 1). In addition, comparing to the ones of WT-ADAMTS13, the distribution of state I of GOF-ADAMTS13 became narrower, while the distribution of state II became broader. These data suggested that both WT-ADAMTS13 and GOF-ADAMTS13 adopted multiple conformational states. We hypothesize that state I represented the “closed” conformation, state III represented the “open” conformation, while the state II represented an intermediate conformation. Five mutations in spacer domain transit more molecules from “closed” conformation to intermediate or “open” conformation.

**Table 1:** The percentages of conformational states of WT- and GOF-ADAMTS13 at pH 7.5.

	state I	state II	state III
WT-ADAMTS13	45.81%	54.19%	N/A
GOF-ADAMTS13	11.78%	75.36%	12.86%

**Lower pH induced ADAMTS13 molecules to extended further.**

**Fig. 4:**
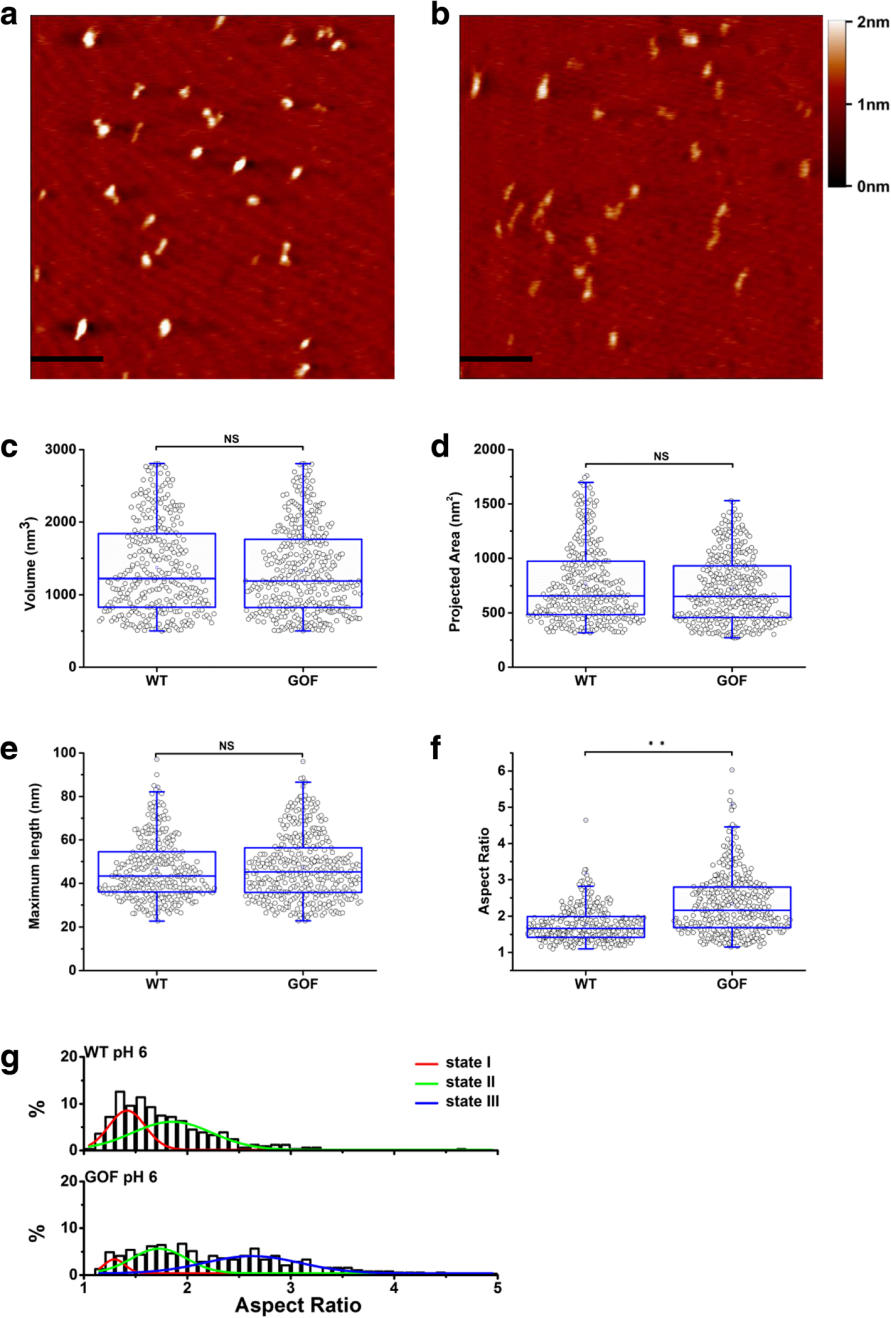
ADAMTS13 molecules further extended at pH 6. AFM images of purified WT-ADAMTS13 (**a**) and GOF-ADAMTS13 (**b**) at pH 6. The horizontal scale bar is 200 nm. The vertical scale bar indicates the height. Volume (**c**), projected area (**d**) maximum length (**e**) and aspect ratio distribution (**f**) analysis of WT-ADAMTS13 (*n* = 333) and GOF-ADAMTS13 (*n* = 393) at pH 6. (**g**) The histogram of aspect ratio of WT-ADAMTS13 and GOF-ADAMTS13. The histogram of WT-ADAMTS13 was well fitted with two Gaussian distributions. The histogram of GOF-ADAMTS13 was well fitted with three Gaussian distributions.

In addition to five mutations in spacer domain, pH was reported to alter the proteolytic activity of ADAMTS13 as well[13, 19]. However, it is not clear whether pH changes the conformation of ADAMTS13. To address this question, WT- and GOF-ADAMTS13 molecules were pre-treated at pH 6, and imaged subsequently (Fig. 4 a and b). Like so, at pH 7.5, the volume of WT- (1370.2 ± 631.8 nm^3^) and GOF-ADAMTS13 (1332.5 ± 597.3 nm^3^) exhibited no significant difference at pH 6 condition (Fig. 4c). In addition, when these volume data were compared to the ones obtained at pH 7.5, no significant difference was observed (Additional file 1 Table S1). The projected area exhibited no significant difference between WT- and GOF-ADAMTS13 neither (762.8 ± 352.3 nm^2^ vs 717.1 ± 307.5 nm^2^, Fig. 4d and Table S2) at pH 6. However, the projected area at pH 6 was remarkably smaller than the one at pH 7.5 for GOF-ADAMTS13 (717.1 ± 307.5 nm^2^ vs 801.9 ± 355.9 nm^2^ Additional file 1 Table S2), but no for WT-ADAMTS13 (762.8 ± 352.3 nm^2^ vs 835.6 ± 264.7 nm^2^, Additional file 1 Table S2). The maximum length of WT-ADAMTS13 at pH 6 was 46.6 ± 14.1 nm, indistinguishable with the one (47.3 ± 14.8 nm) of GOF-ADAMTS13 (Fig. 4e), but significantly longer than the one of WT-ADAMTS13 at pH 7.5 (Additional file 1 Table S3), indicating lower pH extended WT-ADAMTS13 molecules. The aspect ratio of GOF-ADAMTS13 remarkably increased comparing to the one of WT-ADAMTS13 (Fig. 4f). Interestingly, the aspect ratio of both WT- and GOF-ADAMTS13 increased significantly at pH 6 comparing to the counterparts at pH 7.5 (Additional file 1 Table S4), indicating ADAMTS13 molecules became longer and thinner at pH 6.

The distributions of aspect ratio of WT- and GOF-ADAMTS13 at pH 6 were plotted to further analyze the conformational states (Fig. 4g). WT-ADAMTS13 exhibited a two-state distribution while GOF-ADAMTS13 exhibited a three-state distribution, similar to the observations at pH 7.5 (Fig. 3g ). Comparing to WT-ADAMTS13, the percentage of state I of GOF-ADAMTS13 reduced 30.11% (from 38.95 to 8.84%, Table 2), and the percentage of state II reduced 20.12% (from 61.05 to 40.93%, Table 2), contrast to the observation at pH 7.5 (Fig. 3g). When the ratio of states of WT-ADAMTS13 at different pH were compared, the percentage of state I decreased and the one of state II increased at pH 6 (Fig. 3g and 4g, Tables 1 and 2). Notably, the percentage of GOF-ADAMTS13 molecules in state III remarkably increased at pH 6 (from 12.86 to 50.23%, Tables 1 and 2), concomitant with the reduction of the percentages of both state I and states II (from 11.78 to 8.84% and from 75.36 to 40.93% respectively, Tables 1 and 2).

**Table 2:** The percentages of conformational states of WT- and GOF-ADAMTS13 at pH 6.

	state I	state II	state III
WT-ADAMTS13	38.95%	61.05%	N/A
GOF-ADAMTS13	8.84%	40.93%	50.23%

Together, these data indicated that lower pH induced the extension of ADAMTS13 molecules.

## Discussion

ADAMTS13 cleaves the cryptic peptide bond Tyr1605-Met1606 in VWF-A2 domain converting the prothrombotic ULVWF to smaller and less adhesive multimers, preventing aberrant platelet aggregation and thrombus[28, 29]. Deficiency of ADAMTS13 activity resulting from hereditary or acquired etiologies results in TTP[5, 29]. It has been shown that ADAMTS13 adopts a “closed” conformation through the interaction between the spacer and CUB domains[13, 14]. The engagement of VWF D4-CK or antibodies to CUB domains disrupts the spacer-CUB interaction resulting in opening ADAMTS13, and thus increasing the proteolytic activity of ADAMTS13[13–15]. However, conformationally active ADAMTS13 is capable of proteolysing the Aα chain of fibrinogen[17]. South et al. proposed that conformational quiescence of ADAMTS13 prevents proteolytic promiscuity[17]. In addition, a recombinant ADAMTS13 drug (BAX 930) was developed and used in clinical trial (phase I) to treat the congenital form of TTP[30, 31]. Therefore, insights into the conformation of ADAMTS13 advance the understanding of the mechanisms underlying the cleavage of VWF by ADAMTS13, and facilitate recombinant ADAMTS13 drug design.

To investigate the conformation of ADAMTS13, recombinant WT- and GOF-ADAMTS13 were purified from HEK293T cell line, and imaged with AFM which is widely used in imaging biological samples in air[32–34] or liquid[35, 36] and manipulating biological samples[3, 37, 38]. The molecular weights of both WT- and GOF-ADAMTS13 were detected between 130 kDa and 250 kDa (Fig. 1b), consistent with the previous studies indicating that the apparent molecular weight of ADAMTS13 is approximately 190 kDa[25, 39]. By imaging four different proteins whose molecular weights range from 30 kDa to 190 kDa (Fig. 1c), the AFM system was proven to be reliable, supported by the evidence that the volume versus molecular weight plot was well fitted into a straight line (Fig. 1d). Volume analysis is a robust and reliable method which has been used to obtain the stoichiometries of protein-protein assemblies and association constants[26, 27]. No significant difference was observed in both the volume and aspect ratio between purified WT-ADAMTS13 and commercial ADAMTS13 (Fig. 2b and c), indicating that the purified ADAMTS13 folds correctly. In addition, the adhesion frequencies of WT- and GOF-ADAMTS13 binding to VWF-A2 domain were significantly higher than the ones of controls (Fig. 2d). The adhesion frequency of GOF-ADAMTS13 binding to VWF-A2 domain was slightly higher than the one of WT-ADAMTS13 binding to VWF-A2 domain (Fig. 2d), which is consistent with the higher activity of GOF-ADAMTS13 than WT-ADAMTS13[13, 39]. Together, these data demonstrated that the AFM system is reliable to image purified ADAMTS13 molecules which fold correctly and are biologically functional.

To investigate the conformation of WT- and GOF-ADAMTS13, we analyzed volume, maximum length, projected area and aspect ratio of these two types of molecules (Fig. 3 and Fig. 4). No significant difference on the volume was observed between WT- and GOF-ADAMTS13 at different pH as expected (Fig. 3c and 4c, Table 1). The average maximum length of WT-ADAMTS13 was 43.4 ± 8.7 nm (Fig. 3d), which is longer than the one (~ 23 nm) obtained by EM and small angle X-ray scattering[14]. The discrepancy might be due to the influence of AFM tip geometry[40, 41].

Previous findings demonstrated that different conformations of ADAMTS13 with condensed and elongated forms were observed by quick-freeze deep-etch EM[14]. In addition, Roose et al. reported that the ADAMTS13 in healthy donors did not bind to the anti-ADAMTS13 antibody (1C4), which specifically recognizes the “open” conformation of ADAMTS13. In contrast, ADAMTS13 of acute acquired TTP (aTTP) patients bound to 1C4 in 92% of the cases, indicating the conformation of ADAMTS13 is “open” during an acute TTP episode[21]. The authors proposed that an “open” conformation of ADAMTS13 is a hallmark of acute aTTP. In addition, they observed that ADAMTS13 of acute aTTP patients bound to 1C4 with various efficiency and suspected that different types of open conformations of ADAMTS13 exist during acute aTTP. Indeed, the aspect ratio distributions of WT- and GOF-ADAMTS13 molecules demonstrated that WT-ADAMTS13 molecules exhibited two distinct conformational states (state I and state II), while GOF-ADAMTS13 molecules exhibited three distinct conformational states (state I, sate II and state III) at both pH 7.5 and pH 6 conditions (Fig. 3g and Fig. 4g). State III is ought to be the “open” conformation, as this state lacks in WT-ADAMTS13 [13, 14]. The state I with the lowest aspect ratio value is ought to be the “closed” conformation. We propose that state II is an intermediate state. Three linker regions in the distal domains of ADAMTS13 were reported to be responsible for the flexibility of ADAMTS13[16]. The intermediate state might result from the fluctuation of the flexible distal domains of ADAMTS13. Alternatively, the intermediate state might attribute to the transient or partial opening of the conformation of ADAMTS13.

It was reported that low pH increased the activity of ADAMTS13[14, 18, 19]. To investigate if low pH induces an “open” ADAMTS13 conformation, WT- and GOF-ADAMTS13 were pre-treated at pH 6 and imaged with AFM (Fig. 4a and b). The average aspect ratio of both WT- and GOF-ADAMTS13 at pH 6 were significantly higher than the counterparts at pH 7.5 (Additional file 1 Table S4), indicating the molecules were further elongated at pH 6. Similar to the observation at pH 7.5, only two distinct states were observed at pH 6 for WT-ADAMTS13 (Fig. 4g). However, more molecules adopted the intermediate state at pH 6 (61.1% at pH 6 vs 54.2% at pH 7.5, Fig. 4g and 3g, and Tables 1 and 2). It was suggested that the interactions between distal and proximal domains of ADAMTS13 were disrupted resulting in the “open” conformation[14]. However, the percentage of state III at pH 6 dramatically increased for GOF-ADAMTS13 (50.2% at pH 6 vs 12.9% at pH 7.5, Fig. 4g and 3g, and Tables 1 and 2). Furthermore, the difference of aspect ratio of GOF-ADAMTS13 at pH 6 and pH 7.5 was significant (Additional file 1 Table S4), indicating the molecules were elongated further, even though they were “open”. These data suggested that in addition to inducing tertiary structure changes, lower pH might also disrupt intra-domain interactions, increasing the flexibility of ADAMTS13, forming a longer and thinner structure.

## Conclusions

The conformational states of WT- and GOF-ADAMTS13 at different pH were investigated by AFM imaging in the present study. The data demonstrated that both WT- and GOF-ADAMTS13 exist multiple conformational states. Interestingly, in addition to “closed” and “open” states which reported previously, an intermediate state was observed for both WT- and GOF-ADAMTS13. In addition, the conformations of both WT- and GOF-ADAMTS13 were elongated at pH 6, suggesting lower pH might alter the tertiary structure and/or disrupt the intra-domain interactions, which increases the flexibility of ADAMTS13 molecules.

## Additional file

Additional file 1: **Table S1.** One-way ANOVA of volume in different conditions, **Table S2**. One-way ANOVA of projected area in different conditions, **Table S3**. One-way ANOVA of maximum length in different conditions, **Table S4**. One-way ANOVA of aspect ratio in different conditions, **Figure S1**. Histogram of the volume of four different proteins. (DOCX 94 kb)

## Abbreviations.

ADAMTS13: A disintegrin and metalloprotease with a thrombospondin type 1 motif 13
AFM: Atomic force microscopy.
aTTP: Acquired TTP.
BSA: Bovine serum albumin.
BCA: Bicinchoninic acid.
C: Cysteine-rich domain.
CUB: Complement components C1r/C1s, epidermal growth factor-related sea urchin protein, and bone morphogenetic protein-1.
D: Disintegrin domain.
DMEM: Dulbecco’s Modified Eagle Medium.
FBS: Fetal bovine serum.
GOF-ADAMTS13: Gain-of-function ADAMTS13.
M: Metalloprotease domain.
MDTCS: A truncated mutant of ADAMTS13
Opti-MEM: Improved Minimal Essential Medium
PBS: Phosphate buffered saline
S: Spacer domain
SDS-PAGE: Sodium dodecyl sulfate polyacrylamide gel electrophoresis
TSP-1: Thrombospondin type-1 motif
TTP: Thrombotic thrombocytopenic purpura
ULVWF: Ultra-large Von Willebrand factor
VWF: Von Willebrand factor
WT-ADAMTS13: Wild type ADAMTS13

## References

[1] Zheng XL (2013). Structure-function and regulation of ADAMTS-13 protease. J Thromb Haemost..

[2] Dong JF (2007). Structural and functional correlation of ADAMTS13. Curr Opin Hematol..

[3] Wu T, Lin J, Cruz MA (2010). Dong JF Zhu C Force-induced cleavage of single VWFA1A2A3 tridomains by ADAMTS-13. Blood..

[4] Sadler JE (2017). Pathophysiology of thrombotic thrombocytopenic purpura. Blood..

[5] Saha M (2017). McDaniel JK Zheng XL Thrombotic thrombocytopenic purpura: pathogenesis, diagnosis and potential novel therapeutics. J Thromb Haemost..

[6] Feng Y, Li X, Xiao J, Li W, Liu J, Zeng X (2016). ADAMTS13: more than a regulator of thrombosis. Int J Hematol..

[7] Joly BS (2017). Coppo P Veyradier A Thrombotic thrombocytopenic purpura. Blood..

[8] Zheng XL (2015). ADAMTS13, lucky to have a hydrophobic pocket. Blood..

[9] Liu C, Zhao L, Zhao J, Xu Q (2017). Song Y Wang H Reduced ADAMTS-13 level negatively correlates with inflammation factors in plasma of acute myeloid leukemia patients. Leuk Res..

[10] Warlo EMK, Pettersen AR (2017). Arnesen H Seljeflot I vWF/ADAMTS13 is associated with on-aspirin residual platelet reactivity and clinical outcome in patients with stable coronary artery disease. Thromb J..

[11] Gragnano F, Sperlongano S, Golia E, Natale F, Bianchi R, Crisci M (2017). The Role of von Willebrand Factor in Vascular Inflammation: From Pathogenesis to Targeted Therapy. Mediators Inflamm..

[12] Pickens B, Mao Y, Li D, Siegel DL, Poncz M, Cines DB (2015). Platelet-delivered ADAMTS13 inhibits arterial thrombosis and prevents thrombotic thrombocytopenic purpura in murine models. Blood..

[13] South K, Luken BM, Crawley JT, Phillips R, Thomas M, Collins RF (2014). Conformational activation of ADAMTS13. Proc Natl Acad Sci U S A..

[14] Muia J, Zhu J, Gupta G, Haberichter SL, Friedman KD, Feys HB (2014). Allosteric activation of ADAMTS13 by von Willebrand factor. Proc Natl Acad Sci U S A..

[15] South K (2017). Freitas MO Lane DA A model for the conformational activation of the structurally quiescent metalloprotease ADAMTS13 by von Willebrand factor. J Biol Chem..

[16] Deforche L, Roose E, Vandenbulcke A, Vandeputte N, Feys HB, Springer TA (2015). Linker regions and flexibility around the metalloprotease domain account for conformational activation of ADAMTS-13. J Thromb Haemost..

[17] South K (2016). Freitas MO Lane DA Conformational quiescence of ADAMTS-13 prevents proteolytic promiscuity. J Thromb Haemost..

[18] Muia J, Gao W, Haberichter SL, Dolatshahi L, Zhu J, Westfield LA (2013). An optimized fluorogenic ADAMTS13 assay with increased sensitivity for the investigation of patients with thrombotic thrombocytopenic purpura. J Thromb Haemost..

[19] Kokame K, Nobe Y, Kokubo Y (2005). Okayama A Miyata T FRETS-VWF73, a first fluorogenic substrate for ADAMTS13 assay. Br J Haematol..

[20] Di Stasio E, Lancellotti S, Peyvandi F, Palla R (2008). Mannucci PM De Cristofaro R Mechanistic studies on ADAMTS13 catalysis. Biophys J..

[21] Roose E, Schelpe AS, Joly BS, Peetermans M, Verhamme P, Voorberg J (2018). An open conformation of ADAMTS-13 is a hallmark of acute acquired thrombotic thrombocytopenic purpura. J Thromb Haemost..

[22] Lin J, Countryman P, Buncher N, Kaur P, Longjiang E, Zhang Y (2014). TRF1 and TRF2 use different mechanisms to find telomeric DNA but share a novel mechanism to search for protein partners at telomeres. Nucleic Acids Research..

[23] Lin J, Countryman P, Chen H, Pan H, Fan Y, Jiang Y (2016). Functional interplay between SA1 and TRF1 in telomeric DNA binding and DNA–DNA pairing. Nucleic Acids Research..

[24] Levy R, Maaloum M (2002). Measuring the spring constant of atomic force microscope cantilevers: thermal fluctuations and other methods. Nanotechnology..

[25] Fujikawa K, Suzuki H (2001). McMullen B Chung D Purification of human von Willebrand factor-cleaving protease and its identification as a new member of the metalloproteinase family. Blood..

[26] Yang Y (2003). Wang H Erie DA Quantitative characterization of biomolecular assemblies and interactions using atomic force microscopy. Methods..

[27] Kaur P, Wu D, Lin J, Countryman P, Bradford KC, Erie DA (2016). Enhanced electrostatic force microscopy reveals higher-order DNA looping mediated by the telomeric protein TRF2. Sci Rep..

[28] De Caterina R, Madonna R (2016). Von Willebrand factor, ADAMTS13, and coronary microvascular obstruction: beautiful hypotheses, ugly facts. Cardiovasc Res..

[29] South K, Lane DA (2018). ADAMTS-13 and von Willebrand factor: a dynamic duo. J Thromb Haemost..

[30] Scully M, Knobl P, Kentouche K, Rice L, Windyga J, Schneppenheim R (2017). Recombinant ADAMTS-13: first-in-human pharmacokinetics and safety in congenital thrombotic thrombocytopenic purpura. Blood..

[31] Kopic A, Benamara K, Piskernik C, Plaimauer B, Horling F, Hobarth G (2016). Preclinical assessment of a new recombinant ADAMTS-13 drug product (BAX930) for the treatment of thrombotic thrombocytopenic purpura. J Thromb Haemost..

[32] Bonazza K, Rottensteiner H, Schrenk G, Frank J, Allmaier G, Turecek PL (2015). Shear-Dependent Interactions of von Willebrand Factor with Factor VIII and Protease ADAMTS 13 Demonstrated at a Single Molecule Level by Atomic Force Microscopy. Anal Chem..

[33] Lin J, Countryman P, Chen H, Pan H, Fan Y, Jiang Y (2016). Functional interplay between SA1 and TRF1 in telomeric DNA binding and DNA-DNA pairing. Nucleic Acids Res..

[34] Lin J, Countryman P, Buncher N, Kaur P (2014). E L, Zhang Y, et al. TRF1 and TRF2 use different mechanisms to find telomeric DNA but share a novel mechanism to search for protein partners at telomeres. Nucleic Acids Res..

[35] Murugesapillai D, Bouaziz S, Maher LJ, Israeloff NE (2017). Cameron CE Williams MC Accurate nanoscale flexibility measurement of DNA and DNA-protein complexes by atomic force microscopy in liquid. Nanoscale..

[36] Eeftens JM, Katan AJ, Kschonsak M, Hassler M, de Wilde L, Dief EM (2016). Condensin Smc2-Smc4 Dimers Are Flexible and Dynamic. Cell Rep..

[37] Marshall BT, Long M, Piper JW, Yago T (2003). McEver RP Zhu C Direct observation of catch bonds involving cell-adhesion molecules. Nature..

[38] Kellermayer MS (2003). Bustamante C Granzier HL Mechanics and structure of titin oligomers explored with atomic force microscopy. Biochim Biophys Acta..

[39] Jian C, Xiao J, Gong L, Skipwith CG, Jin SY, Kwaan HC (2012). Gain-of-function ADAMTS13 variants that are resistant to autoantibodies against ADAMTS13 in patients with acquired thrombotic thrombocytopenic purpura. Blood..

[40] Shen J, Zhang D (2017). Zhang F-H Gan Y AFM tip-sample convolution effects for cylinder protrusions. Applied Surface Science..

[41] Burt DP, Wilson NR, Janus U (2008). Macpherson JV Unwin PR In-situ atomic force microscopy (AFM) imaging: influence of AFM probe geometry on diffusion to microscopic surfaces. Langmuir..

