# Supplementary material for: AFM Imaging Reveals Multiple Conformational States of ADAMTS13
Source: J Biol Eng. 2019 Jan 22;13:9. doi: 10.1186/s13036-018-0102-y (PMC6343300; doi:10.1186/s13036-018-0102-y)
Supplement: Supplementary file 1 — Table S1. One-way ANOVA of volume in different conditions, Table S2. One-way ANOVA of projected area in different conditions, Table S3. One-way ANOVA of maximum length in different conditions, Table S4. One-way ANOVA of aspect ratio in different conditions, Figure S1. Histogram of the volume of four different proteins. (DOCX 94 kb) [file 13036_2018_102_MOESM1_ESM.docx]

**AFM imaging reveals multiple conformational states of ADAMTS13**

Shanshan Yu^1^, Wang Liu^1^, Jinhua Fang^1^, Xiaozhong Shi^1^, Jianhua Wu^1^, Ying Fang^1#^, Jiangguo Lin^1#^

**SUPPLEMENTAL TABLES**

**Table S1. One-way ANOVA of volume in different conditions**

**Table S2. One-way ANOVA of projected area in different conditions**

**Table S3. One-way ANOVA of maximum length in different conditions**

**Table S4. One-way ANOVA of aspect ratio in different conditions**

**SUPPLEMENTAL FIGURE**

**Figure. S1** **Histogram of the volume of four different proteins.**

**Table S1. One-way ANOVA of volume in different conditions**

| **P** | **WT pH 7.5** | **WT pH 6** | **GOF pH 7.5** | **GOF pH 6** |
| --- | --- | --- | --- | --- |
| **WT pH 7.5** |  | **NS** | **NS** | **NS** |
| **WT pH 6** | **NS** |  | **NS** | **NS** |
| **GOF pH 7.5** | **NS** | **NS** |  | **NS** |
| **GOF pH 6** | **NS** | **NS** | **NS** |  |

**Table S2. One-way ANOVA of projected area in different conditions**

| **P** | **WT pH 7.5** | **WT pH 6** | **GOF pH 7.5** | **GOF pH 6** |
| --- | --- | --- | --- | --- |
| **WT pH 7.5** |  | **NS** | **NS** | ****** |
| **WT pH 6** | **NS** |  | **NS** | **NS** |
| **GOF pH 7.5** | **NS** | **NS** |  | ***** |
| **GOF pH 6** | ****** | **NS** | ***** |  |

**Table S3. One-way ANOVA of maximum length in different conditions**

| **P** | **WT pH 7.5** | **WT pH 6** | **GOF pH 7.5** | **GOF pH 6** |
| --- | --- | --- | --- | --- |
| **WT pH 7.5** |  | ***** | ****** | ****** |
| **WT pH 6** | ***** |  | **NS** | **NS** |
| **GOF pH 7.5** | ****** | **NS** |  | **NS** |
| **GOF pH 6** | ****** | **NS** | **NS** |  |

.

**Table S4. One-way ANOVA of aspect ratio in different conditions**

| **P** | **WT pH 7.5** | **WT pH 6** | **GOF pH 7.5** | **GOF pH 6** |
| --- | --- | --- | --- | --- |
| **WT pH 7.5** |  | ****** | ****** | ****** |
| **WT pH 6** | ****** |  | ****** | ****** |
| **GOF pH 7.5** | ****** | ****** |  | ****** |
| **GOF pH 6** | ****** | ****** | ****** |  |


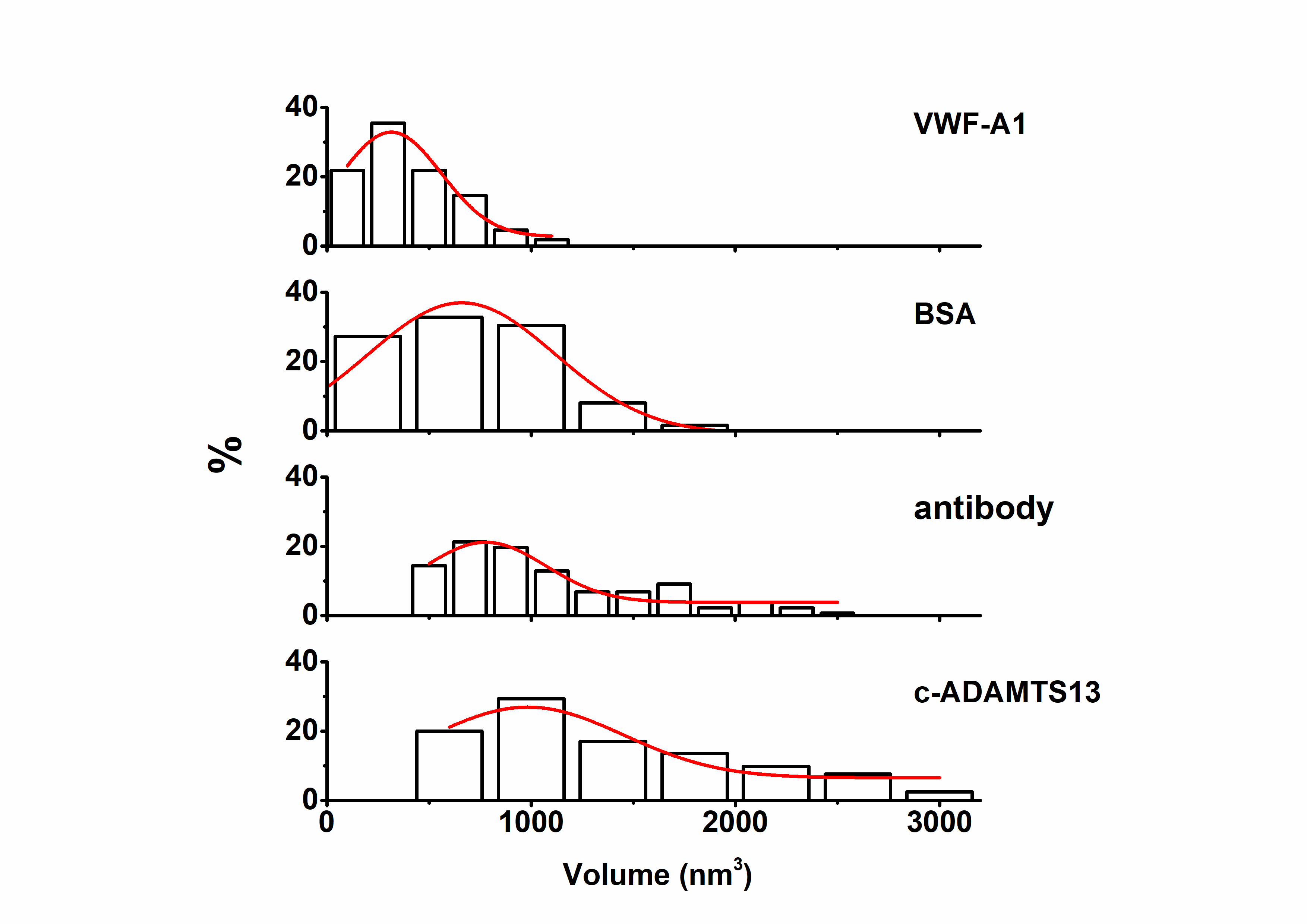


**Figure. S1** **Histogram of the volume of four different proteins.** The histogram of volume of VWF-A1 (30 kDa), BSA (67 kDa), anti-His tag antibody (150 kDa) and commercial ADAMTS13 (190 kDa). The histogram of these four proteins were fitted with Gaussian distribution. Particles whose volume was in the 95% confidence interval (μ ± 1.96σ) were selected for further analysis.
